# The FoxOs are in the ApoM house

**DOI:** 10.1172/JCI158471

**Published:** 2022-04-01

**Authors:** MacRae F. Linton, Patricia G. Yancey, Zoe M. Leuthner, Jonathan D. Brown

**Affiliations:** 1 Department of Medicine, Atherosclerosis Research Unit, Division of Cardiovascular Medicine and; 2 Department of Pharmacology, Vanderbilt University School of Medicine, Nashville, Tennessee, USA.

## Abstract

The prevalence of metabolic syndrome continues to increase globally and heightens the risk for cardiovascular disease (CVD). Insulin resistance is a core pathophysiologic mechanism that causes abnormal carbohydrate metabolism and atherogenic changes in circulating lipoprotein quantity and function. In particular, dysfunctional HDL is postulated to contribute to CVD risk in part via loss of HDL-associated sphingosine-1-phosphate (S1P). In this issue of the *JCI*, Izquierdo et al. demonstrate that HDL from humans with insulin resistance contained lower levels of S1P. Apolipoprotein M (ApoM), a protein constituent of HDL that binds S1P and controls bioavailability was decreased in insulin-resistant db/db mice. Gain- and loss-of-function mouse models implicated the forkhead box O transcription factors (FoxO1,3,4) in the regulation of both ApoM and HDL-associated S1P. These data have important implications for potential FoxO-based therapies designed to treat lipid and carbohydrate abnormalities associated with human metabolic disease and CVD.

## FoxO and insulin signaling

The mammalian forkhead box O family of transcription factors (FoxO1, FoxO3, FoxO4, and FoxO6) possess an evolutionarily conserved forkhead box (also called, F-box or winged helix) DNA-binding domain (DBD) along with a nuclear localization signal, a nuclear export sequence, and a C-terminal transactivating domain (1). FoxO1,3,4 are master transcriptional regulators of the insulin/insulin growth factor signaling axis in metabolically active tissues including liver, skeletal muscle, adipose tissue, and heart (2). In response to insulin, FoxOs are phosphorylated by Akt at conserved serine/threonine residues (3). This posttranslational modification promotes the translocation of FoxOs out of the nucleus into the cytoplasm, thereby inactivating them. FoxO1,3,4 control carbohydrate and lipid metabolism during physiologic adaptations to fasting; their dysregulation directly impacts pathologic gene expression caused by insulin resistance, diabetes mellitus, and metabolic syndrome (4).

**Figure 1:**
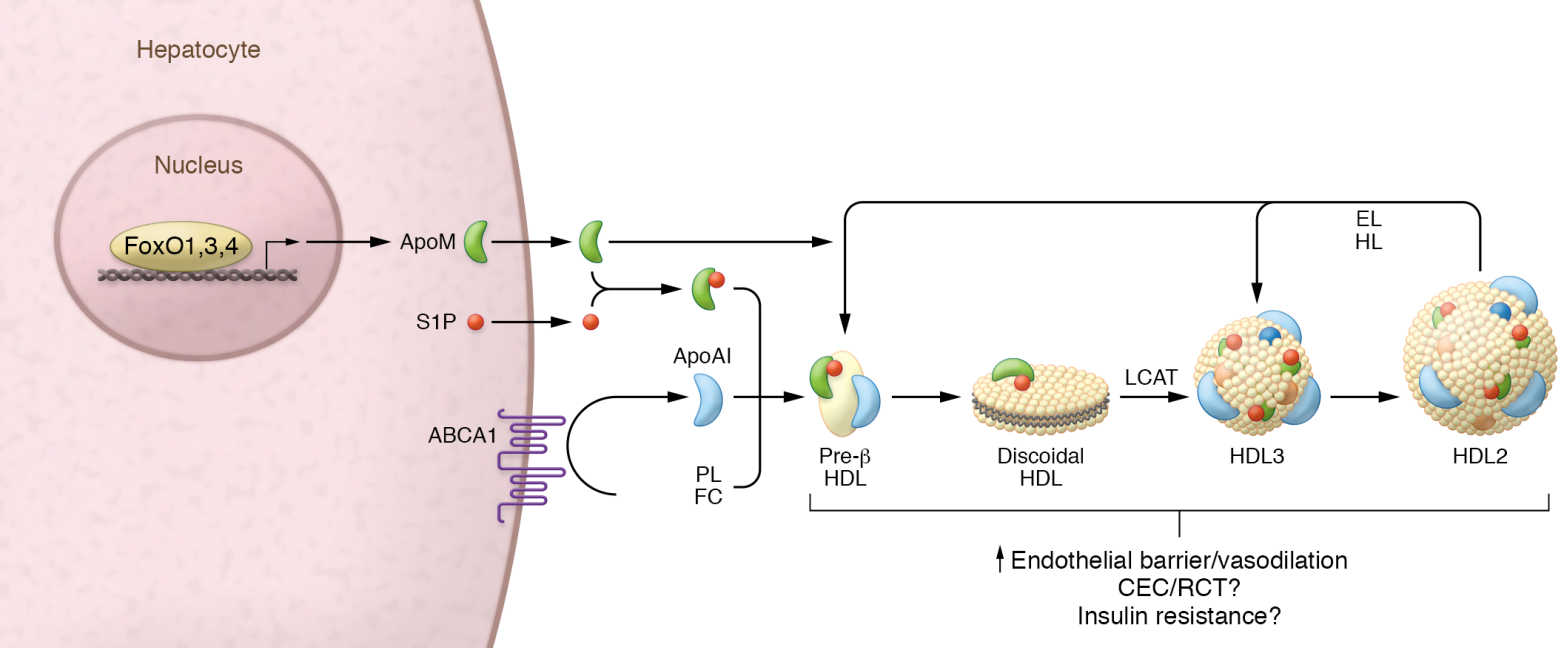
FoxO1,3,4 regulate plasma levels of the HDL-ApoM-S1P complex. Hepatic FoxO transcription factors control the expression of *Apom* by binding to the promoter and enhancer regions of the gene. ApoM is secreted and forms a complex with plasma S1P. The majority of plasma ApoM-S1P associates with HDL and is found to be associated with pre-β and α (HDL2 and HDL3) migrating subpopulations. ApoM may stimulate the formation of pre–β-HDL during the endothelial lipase– (EL-) and hepatic lipase–mediated (HL-mediated) conversion of α-HDL2 to HDL3 and pre–β-HDL. The HDL-ApoM-S1P complex enhances endothelial barrier integrity and vasodilation. The roles for the HDL-ApoM-S1P complex in insulin resistance, HDL CEC, and RCT have not been clearly demonstrated. FC, free cholesterol; LCAT, lecithin-cholesterol acyltransferase; PL, phospholipid.

There are strong pathophysiologic links between insulin resistance, which is a state proposed to result in persistent activation of FoxOs, and dyslipidemia. In this issue of the *JCI*, Izquierdo et al. explored the role of FoxO1,3,4 in the regulation of HDL-associated apolipoprotein M (ApoM) and sphingosine-1-phosphate (S1P) (Figure 1 and ref. 5). Plasma ApoM mainly associates with HDL (6) and interacts with S1P (7, 8). Liver-specific deletion of *Foxo1,3,4* in mice resulted in a 90% reduction in *Apom* mRNA and nearly absent ApoM protein in mouse livers. Of note, the authors targeted *Foxo1,3,4* because of the compensation that can occur from each of these closely related F-box family members. Mechanistic experiments revealed that a constitutively active form of FoxO1 lacking the Akt phosphorylation sites increased *Apom* expression by 40% in primary murine hepatocytes, confirming that FoxO1 induces *Apom* in a cell-autonomous manner (5). FoxOs can also regulate gene transcription through indirect mechanisms (9). Thus, the finding that *Apom* induction required an intact DBD demonstrated that engagement at *cis*-regulatory DNA was required for FoxO1 to control *Apom* expression. The authors then performed ChIP-PCR assays to map FoxO1 occupancy at two promoter and two enhancer sites. Although they were unable to detect changes in FoxO1 signal between chow and high-fat diet treatments, they did detect binding events within all groups (5). We can interpret the absence of dynamic FoxO1 recruitment several ways with respect to transcription control: (a) gain and loss of FoxO1 may occur at de novo sites outside the chosen regions, as recently described in another study in which FoxO1 distribution at promoters and enhancers differed between genes involved in carbohydrate versus lipid metabolism (10); (b) FoxO1 could remain DNA bound, with the integrated transcriptional response governed by recruitment of corepressors or cooperativity between FoxO3,4, FoxA2, or other transcription factors known to colocalize with FoxOs, such as hepatocyte nuclear factor 4 (HNF4), CCAAT/enhancer binding protein β (CEBPβ), estrogen-related receptor α (ERRα), or the glucocorticoid receptor (GR) (11, 12); (c) the high-fat diet may not have induced sufficient stress to alter FoxO1 activation as compared with other diabetic or insulin-resistant states. The results of Izquierdo et al. convincingly demonstrate that *Apom* is a transcriptional target of the FoxO family, but leave unanswered the question about the underlying mechanism for how FoxO function was altered to result in reduced *Apom* transcription in the insulin resistance models.

## Posttranslational modifications influence transcriptional output

One conundrum related to insulin resistance is the degree to which FoxOs are activated or inactivated. Existing models suggest that insulin signaling diverges downstream of its cognate receptor, such that both gain and loss of function of FoxOs can regulate carbohydrate and lipid metabolic pathways. Indeed, beyond Akt phosphorylation, FoxOs can be phosphorylated by kinases (MAPK, JNK, AMPK) in response to stress signals as well as reversibly acetylated and ubiquitylated (1, 13, 14). These diverse posttranslational modifications impact nuclear localization, transcription factor expression levels, DNA binding, transactivation function, and protein-protein interactions, thereby modulating transcriptional output at individual genes. In light of the diverse modifications, the concordant finding that FoxO1,3,4 genetic inactivation and insulin resistance — a state of proposed FoxO activation — resulted in similar reductions in *Apom* mRNA strongly suggest that FoxO-dependent transcription is paradoxically inactivated at the *Apom* locus in the insulin-resistant models tested (db/db, Western diet, and gold thioglucose–induced hypothalamic injury). Notably, hyperinsulinemia can inactivate FoxA2 via site-specific phosphorylation, leading to a decrease in *Apom* expression in obese mice (15). Thus, the data presented by Izquierdo et al. add evidence that hyperinsulinemia associates with inactivation of FoxO family members, decreases ApoM expression, and thereby reduces S1P-bound HDL. These observations agree with studies showing that people with type 2 diabetes have reduced plasma ApoM or S1P levels and that their ApoM levels are inversely correlated with an insulin resistance index (16, 17). Indeed, *Apom^–/–^* mice generated using CRISPR/Cas9 engineering have decreased insulin sensitivity, whereas transgenic mice overexpressing ApoM are less insulin resistant and more glucose tolerant. These changes in insulin sensitivity associate with Akt and AMPK signaling via S1P receptor 1/3 activation (16). In contrast, *Apom^–/–^* mice, generated by inserting a neomycin resistance–encoding cassette in the *ApoM* locus, have increased glucose tolerance (18), while Izquierdo and colleagues demonstrated that rescuing the expression of ApoM in db/db mice failed to augment glucose tolerance (5). In addition, liver-specific knockdown of FoxO1,3,4 in mice decreased the HDL-ApoM-S1P complex, but also decreased hepatic glucose production (5). Thus, the role of the HDL-ApoM-S1P in insulin resistance is controversial in mouse models. In humans, it is unclear whether HDL-ApoM-S1P critically contributes to diabetes development, since several genetic variants in the promoter region of ApoM are not linked to increased diabetes risk (19). Whether FoxO3 and FoxO4 collaborate with FoxO1, as suggested by the genetic model, specifically at the *ApoM* locus and more generally at metabolic genes, will necessitate follow-up studies that examine genomic occupancy and transcriptional cooperativity between these transcription factors.

More broadly considered, these data complicate the links between the FoxO activation state in response to hyperinsulinemia and associated dyslipidemias caused by abnormal regulation of genes involved in lipoprotein synthesis. In particular, the results presented by Izquierdo et al. suggest that binary models of on-and-off states are too simplistic to explain how FoxOs transcriptionally control lipid metabolism in the context of hyperinsulinemia.

## Insight into HDL function

Izquierdo and colleagues (5) demonstrated that hepatic FoxOs control plasma levels of HDL-ApoM-S1P1 in mice, providing insight into HDL function. HDLs protect against coronary artery disease (CAD) by promoting reverse cholesterol transport (RCT) and endothelial integrity and limiting inflammation and oxidation (20). HDLs are heterogenous in size, charge, lipid subspecies, and protein composition (20). Notably, HDL components are continuously regulated and changing. Apolipoprotein AI (ApoAI), along with a wide range of other proteins and diverse lipids, mediates HDL functions. In diseases that confer increased risks for CAD, such as diabetes (17), changes in HDL constituents and oxidative modifications can render HDL dysfunctional, increasing the atherogenic risk. Further, interventions to scavenge reactive dicarbonyls such as isolevuglandins (IsoLGs) and malondialdehyde (MDA) decrease atherosclerosis and improve HDL function (21). Low levels of HDL-S1P have also been linked to cardiovascular disease (CVD) (22). Thus, ApoM-S1P may be key contributors to HDL function.

The HDL-ApoM-S1P complex has been reported to promote endothelial barrier and vasodilation, as well as antioxidant, antiinflammatory, and cholesterol efflux capacity (CEC) (6, 23, 24). S1P signals via GPCRs, and when bound to ApoM, as opposed to albumin, more potently regulates endothelial function (7, 25). In vitro and in vivo studies suggest that HDL-ApoM-S1P protects against endothelial inflammation and promotes barrier integrity and vasodilation (7, 23). Interestingly, Izquierdo and colleagues (5) determined that the flow-mediated vasodilation in insulin-resistant individuals and controls was not associated with HDL or S1P levels. However, as the authors suggest, an association could have been masked by other factors. Indeed, total HDL-ApoM-S1P may not be a measure of endothelial protective effects. HDL from humans with type 2 diabetes versus controls has similar ApoM content, but HDL from those with diabetes is less efficient at preventing endothelial TNF-α expression and activating eNOS, functions that correlate with plasma S1P levels (17). In humans with type 1 diabetes, the ApoM-S1P complex shifts to larger HDLs that provide less protective endothelial function compared with dense HDL-ApoM-S1P from controls (26). Thus, it is plausible that the effects on vascular dilation are linked to dense HDL-ApoM-S1P subpopulations rather than to total HDL-ApoM-S1P. Alternatively, other components of HDL (i.e., ApoAI; ref. 20) or other sources of S1P, such as endothelium-derived S1P (23), may be linked to endothelial function. Izquierdo et al. (5) also demonstrate that the decreased HDL-ApoM-S1P in L-FoxO1,3,4 mice did not change alveolar barrier function. In contrast, other studies showed decreased barrier integrity and vasodilation in *Apom^–/–^* mice and improved endothelial function in mice overexpressing ApoM (7, 23). These contradictory findings may be due to the effects of the HDL-ApoM-S1P on endothelial function being obscured by other factors, such as low glucose production in mice lacking hepatic FoxO1,3,4 (L-FoxO1,3,4 mice) (5).

HDL CEC is independently associated with CVD risk (27). ApoM may impact the formation of pre–β-HDLs, which are efficient cholesterol acceptors of ABCA1 (20). In controls and individuals with type 2 diabetes, plasma ApoM levels predict pre–β-HDL levels (28). In addition, plasma from *ApoM*-transgenic forms more pre–β-HDL ex vivo than does plasma from WT and *Apom^–/–^* mice (24). Pre–β-HDL levels are also decreased in *Apom^–/–^* mice and in *Tcf1^–/–^* and *Foxa2^+/–^* mice, which have decreased ApoM levels (15, 29). Interestingly, Izquierdo et al. (5) demonstrate that HDL from L-FoxO1,3,4 versus WT mice had equally effective CEC in cholesterol-enriched macrophages despite decreased levels of HDL and ApoM. Although the HDL subpopulations were not investigated in L-FoxO1,3,4 mice or compared with those in WT mice, the results were similar to those of other studies showing that HDLs from *Apom^–/–^* versus WT mice have a similar CEC (30). However, another study suggested that HDLs from ApoM-deficient mice have impaired CEC (29). These discrepancies are likely due to differences in HDL isolation and/or CEC assays. In particular, CEC assays in which ABCA1 was not upregulated likely failed to stringently examine the effects of ApoM on HDL CEC (29). Interestingly, HDL from *ApoM*-transgenic mice has enhanced CEC from cholesterol-enriched macrophages when compared with HDL from WT mice, which is consistent with increased pre–β-HDL formation (24, 30). However, HDL from *ApoM*-transgenic mice is enriched in ApoE, which could impact CEC, and the 11-fold higher ApoM levels are probably not physiologically relevant (24). In contrast to mice, HDL and ApoM isolated from humans contain mainly α-migrating particles, which have an enhanced CEC compared with total HDL and HDL particles devoid of ApoM (6). However, total HDL and HDL devoid of ApoM have a similar CEC, which is consistent with HDL and ApoM comprising only 5% of total HDL (6). Nonetheless, we postulate that, while ApoM is not critical to CEC, it may augment pre–β-HDL formation in the arterial wall. However, in vivo RCT studies with mice demonstrated that ApoM does not modulate the flux of cholesterol from cholesterol-enriched macrophages to the liver for excretion (8, 30). Thus, roles for ApoM in modulating HDL, CEC, and RCT have yet to be clearly elucidated and require further investigation.

## Version 1 04/01/2022

Electronic publication
